# Neural bottlenecks: axon count, distribution, and conduction in the *Manduca sexta* neck connective

**DOI:** 10.1007/s00359-025-01755-4

**Published:** 2025-08-20

**Authors:** Leo Wood, Karrah Hayes, Varun Sharma, Eric Sun, Max Chen, Simon Sponberg

**Affiliations:** 1 Quantitative Biosciences Program, Georgia Institute of Technology, Atlanta, GA 30313 USA; 2 School of Physics, Georgia Institute of Technology, Atlanta, GA 30313 USA; 3 Living Dynamical Systems Vertically Integrated Project Team, Georgia Institute of Technology, Atlanta, GA 30313 USA; 4 Coulter Department of Biomedical Engineering, Georgia Institute of Technology, Atlanta, GA 30313 USA; 5 School of Biological Sciences, Georgia Institute of Technology, Atlanta, GA 30313 USA

**Keywords:** Neuroanatomy, Conduction velocity, Neural bottlenecks, Ensheathing glia

## Abstract

Large flying insects precisely control fast maneuvers, a demanding task made more difficult by the limitation that all information between the brain and body is transmitted through a single transmission line, the neck connective. Despite this neuroanatomical structure constraining both the amount and timing of all information between the brain and body, little is known about how severe these bottlenecks are. We sought to understand this structure in the hawkmoth *Manduca sexta* by directly measuring axon count and conduction velocities in their neck connective, using a nanometer-scale complete map of the neck connective in concert with microelectrode array recordings from hundreds of neurons. We hypothesized that *Manduca* opts for a large spatial bottleneck, with comparatively few neurons in their neck connective compared to their brain size, but latency constraints of agile flight necessitate adaptations for increased conduction velocity compared to small insects. *Manduca* had 8,874 total neck connective axons, a number similar to fruit flies despite *Manduca*’s order of magnitude greater body and brain size. Yet *Manduca* had far more giant axons, and the average conduction velocity of those axons exceeded 2 m/s, indicating a strong pressure on reducing neck connective latency. Both ascending and descending units were equally fast, and analyzing how velocity scales with diameter suggested adaptations beyond just axon size are increasing velocity. This data indicates *Manduca*’s neck connective faces similar requirements to other species in terms of number of neurons, but more acute pressures for higher conduction velocity and reduced latency in the neck connective.

## Introduction

In all bilaterian animals, the nervous system is structured as a nerve cord, with the brain connected to peripheral ganglia interspersed along the length of the cord (Arendt et al. 2016). As a result of this structure, a cable of axons on each side of any bilaterian animal, running through the neck, carries all neural activity between the brain and the body of the animal. These tracts of axons, two distinct hemiconnectives (one on each side) which together we refer to as the neck (or cervical) connective, form a bottleneck through which all central and peripheral sensory, motor, and command neural information has to be exchanged. Outside of slow-moving blood- or hemolymph-carried neuropeptides, there is no way for the nervous system to bring information into and out of the brain except via this one tract. Thus the neck connective forms what we will call a “spatial” and “temporal” bottleneck: Spatially, the number of neurons in the connective constrains the amount of information that can be transmitted, and temporally, the conduction velocity of those neurons bound the latency (travel time) of that information. Increasing either of these bounds via more or faster neurons is energetically costly (Attwell and Laughlin 2001; Laughlin et al. 1998), so animals must strike some balance of these factors. How pronounced are these bottlenecks? Specifically, how many neurons are actually in a given animal’s neck connective, and how fast can these neurons carry information?

Despite its importance, quantifying the counts and size of neurons of the neck connective is challenging. Outside of *Drosophila melanogaster*’s recent peripheral connectome projects (Scheffer et al. 2020; Stürner et al. 2025; Takemura et al. 2023), there are few bilaterian animals where both of these questions can be answered with high confidence. What is known in *Drosophila* indicates that the neck connective is on the order of 3700 axons (Marin et al. 2023; Stürner et al. 2025). With a brain containing 150,000–200,000 neurons (Raji and Potter 2021; Scheffer et al. 2020), this indicates 40–50$\times$ fewer neurons than the brain in the neck connective, a ratio that certainly indicates compression, but not necessarily a limiting bottleneck (as that many fewer neurons may be all that is needed for the neck connective). Temporal latencies through the *Drosophila* neck connective vary based on axon diameter, with absolute maximum conduction velocities carried via the giant fibers, two $\sim$8 ${\upmu }$m diameter axons that mediate escape responses (Wyman et al. 1984). These axons carry action potentials down the $\sim$220 ${\upmu }$m long neck connective (Namiki et al. 2018a) at 1–2 m/s (Kadas et al. 2019; Wyman et al. 1984), leading to a minimum neck connective latency of 0.1$-$0.2 ms. While most other neck connective axons are on the order of 5–10$\times$ smaller than the giant fibers and thus conduct action potentials much slower, the short length of *Drosophila*’s neck connective means that for latencies to be less than a single 5 ms wingbeat even slow conduction velocities on the order of 0.04 m/s are sufficient. *Drosophila*, then, does not seem to be particularly limited in information transfer by its neck connective.

However, larger insects may quickly start facing transmission challenges. *Drosophila*’s small size means that information bottlenecks and energetic penalties are greatly reduced; no long, expensive axons, a much smaller brain, and very little latency even at slow conduction velocities. In larger flying insects with much larger brains, connective tracts are many times longer than *Drosophila*’s entire body. Hawkmoths such as *Manduca sexta* have adult bodies longer than 10 cm, with neck connectives on the order of 1 cm in length. Despite this size, *Manduca* and other hawkmoths are incredibly agile, capable of hovering and feeding mid-air from targets moving at 14 Hz (Roth et al. 2016). They also boast some of the highest forward flight speeds (5–8 m/s) of any insect (Menz et al. 2022; Stevenson et al. 1995). The energetic cost of a neuron scales with the surface area of its membrane (Laughlin et al. 1998), so the long connective tracts of insects like *Manduca* greatly heighten the energetic cost of having more neurons, or making those neurons larger in diameter to conduct faster. But these larger insects also seem to have greater spatial and temporal demands on their connectives: Larger sensory and motor structures have increased information capacity requirements (Chittka and Niven 2009), and their flight agility suggests heightened requirements for fast conduction. Information capacity, at least can be improved via firing rate, spike timing precision, or better coding schemes (Borst and Theunissen 1999; Stevens and Zador 1995), but the temporal bottleneck necessitates higher conduction velocity. For these reasons, we hypothesize that: (1) Large flying insects like *Manduca* have a large spatial bottleneck, opting for far fewer connective neurons than their larger brains would suggest, and (2) These insects reduce their temporal bottleneck with greatly enhanced conduction velocities, resulting in adaptations for velocity increase such as giant axons.

The first of these hypotheses, regarding the neck connective’s spatial bottleneck, has been partially developed by study of descending neurons in species such as cockroaches, dragonflies, and crickets using dye backfill labeling (Okada et al. 2003; Staudacher 1998; Severina et al. 2016). In Lepidoptera, backfills were utilized in the silkmoth *Bombyx mori* to systematically characterize the morphology and anatomy of descending neurons (Namiki et al. 2018b), and similarly in the noctuid *Helicoverpa armigera* (Liu et al. 2023). Comparison across all of these studies with *Drosophila* indicates that descending neurons are highly homologous across insects in structure and number (Hsu and Bhandawat 2016). But while these backfill studies are crucial for understanding the spatial bottleneck of the insect neck connective, they are limited to only observing *descending* neurons, lacking a complete picture of the connective. To fully understand the spatial bottleneck, a *complete* count of neck neurons, including those much smaller than 1 ${\upmu }$m in diameter, is needed. Electron microscopy is sufficient for such a task, and has been applied before towards complete neck connective surveys. Outside of the *Drosophila* connectomes, electron microscope mosaics were used to estimate the number of neurons in connectives of the desert locust *Schistocerca gregaria*, with an estimated $\sim$3143 axons in one hemiconnective (one half of the total neck connective) (Rowell and Dorey 1967). Certainly high-resolution electron microscopy has been applied to hawkmoths outside of the neck connective, including imaging of the abdominal nerve cord (Pichon et al. 1972) and prothoracic ganglion (Cantera 1993). Qualitative observation of large-scale ultrastructure and giant ($>10$ ${\upmu }$m diameter) axons has been performed using electron microscopy in large flying insects such as locusts (Rowell and Dorey 1967; Williamson and Burns 1982), houseflies, blowflies (Coggshall et al. 1973), and even *Manduca* (Kanzaki et al. 1991). But outside of *Drosophila* and *Schistocerca*, no insect has combined high resolution and complete coverage to survey the neck connective of *all* axons.

The second of these hypotheses, regarding the neck connective’s temporal bottleneck, has fewer answers from prior study. To measure latency, one needs to measure conduction velocity down the neck connective. But conduction velocity has never been measured in the neck connective of *Manduca* or other hawkmoths to the best of our knowledge. Estimates of large axon diameters are available, and conduction velocity has long been understood to scale with axon diameter. This scaling is agreed to be linear in vertebrate axons with myelin, a many-layered glial sheath which increases conduction velocity (Rushton 1951; Waxman and Bennett 1972). Unmyelinated axons, however, have a less clear scaling. Certainly conduction velocity and diameter are related in all axons, and from basic cable theory should be related by a square root without myelin ($V=d^{0.5}$) (Hodgkin 1954; Rushton 1951). Yet actual measurements of unmyelinated axons usually find exponents higher than 0.5, such as 0.7$-$0.8 in cockroach motor neurons (Pearson et al. 1970) or near linear in unmyelinated cat hindlimb axons (Hoffmeister et al. 1991). Conduction velocity in unmyelinated axons can vary independently of diameter due to ion channel density, rate constants, and membrane voltages to scale predictably across tissues (Castelfranco and Hartline 2015; Waxman 1975). While there are some behavioral indications of latency such as the 300 ms delay between stimulus and flight initiation (Manjunath et al. 2024), these are not sufficient to determine true neck connective conduction velocity or latency.

**Fig. 1:**
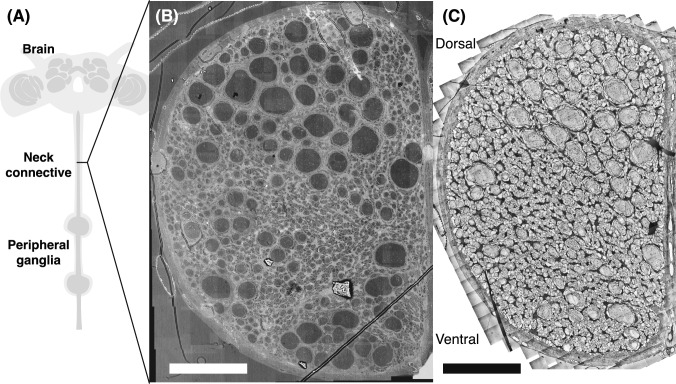
Complete high-resolution EM mosaics of *Manduca* neck hemiconnective (one lateral side of neck connective). **A** Diagram of *Manduca*’s nervous system, with the neck connective identified. **B** SEM image of neck hemiconnective of *Manduca sexta*, scale bar is 50 ${\upmu }$m. 21,360$\times$15,723 pixel image was acquired in single pass at a resolution of 12.28 nm/pixel. No postprocessing was performed. **C** TEM mosaic of neck hemiconnective, acquired from a different individual moth than (**B**). Scale bar is 50 ${\upmu }$m. Image is scaled the same as the SEM scan in (**B**). 452 individual 1980$\times$2024 pixel TEM images, acquired at a resolution of 5.1 nm/pixel. As described in methods, persistent darkness gradients are corrected from each image and an adaptive histogram equalization performed before stitching

In this work we combine a complete, nanometer-scale neuroanatomical map of the neck connective of the hawkmoth *Manduca sexta* (Fig. 1) with extracellular sampling of axon conduction velocity. Utilizing a mosaic of 452 transmission electron microscope (TEM) images, we perform a complete count of the number, location, and diameters of every axon in *Manduca*’s neck connective, as well as identifying unique ultrastructural features such as many-layered ensheathing glia. In concert with this microscopy, we apply multi-shank microelectrode array recordings from the *Manduca* neck connective to determine conduction velocity and latency of several hundred axons. From both these sets of measurements, we are able to quantify the degree to which the neck connective in the large, agile hawkmoth *Manduca* actually serves as a spatial and temporal bottleneck.

## Methods

### Animals

For all experiments male and female *Manduca sexta* pupae were reared into adults in incubators at $25\,^{\circ }$C, housed communally on a 12-h light–dark cycle. Microscopy was performed on *Manduca* obtained from the Case Western Reserve University colony, while conduction velocity measurements were conducted on individuals obtained from a colony at the University of Washington, and a recent colony at the Georgia Institute of Technology seeded using moths from the University of Washington colony.

### Sample collection and preparation

*Manduca sexta* moths were cold-anesthetized for 30 min before the neck region was descaled using pressurized air. Neck connectives, cut at the base of the brain and entrance to the thorax, were placed into 2.5% glutaraldehyde in 0.1 M cacodylate, pH 7.4, in labeled vials. Samples were fixed for $\sim$11 h then transferred to 0.1 M cacodylate buffer for transport to the Emory University Integrated Electron Microscopy Core Facility for processing.

To prepare for TEM imaging, samples were washed in 0.1 M cacodylate buffer, post fixed in 1% buffered osmium tetroxide for 90 min then washed in de-ionized water. This was followed by dehydration in an ascending ethanol series ending in four changes of dry 100% ethanol. The samples were infiltrated overnight with a 1:1 mixture of ethanol and Eponate $12^{\textrm{TM}}$ epoxy resin. Infiltration with epoxy resin continued with 100% resin all day ($\sim$6 h) and fresh resin all night ($\sim$10 h). The samples were embedded in fresh resin in labeled $\hbox {Beem}^{\textrm{TM}}$ capsules the next day and polymerized for three days at $6\,^{\circ }$C.

Ultrathin (70–80 nm) sections were cut using a $\hbox {Leica}^{\textrm{TM}}$ Ultracut 6 ultramicrotome and a $\hbox {Diatome}^{\textrm{TM}}$ diamond knife. The sections were collected onto 100 mesh and single hole (400 ${\upmu }$m diameter) EM Sciences copper grids with carbon stabilized $\hbox {Formvar}^{\textrm{TM}}$ support films. Sections were post-stained with 5% Uranyl Acetate and Reynold’s Lead Citrate stains.

### Electron microscopy

**Fig. 2:**
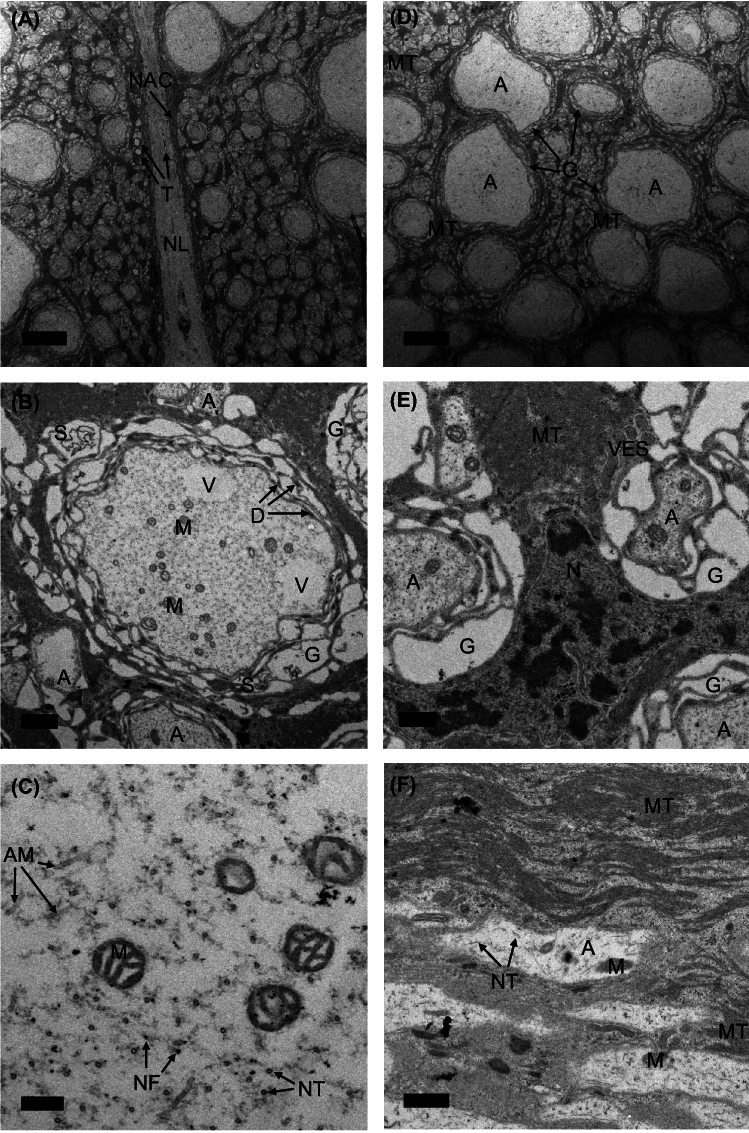
TEM images of interest from *Manduca sexta* neck connective. **A** Transverse section demonstrating the two separate hemiconnective tracts, the neural lamella (NL) that surrounds them, and the internal layer of non-axonal cells (NAC). Tracheoles (T) are found within the lamella and the connectives. Scale bar: 10 ${\upmu }$m. **B** Transverse section of a medium-large axon. A, axon; G, ensheathing glia; M, mitochondria; V, vacuoles; D, desmosomes; S, chromatin strand. Scale bar: 1 ${\upmu }$m. **C** Magnified structures within transverse section of axoplasm. AM, actin microfilaments; NF, neurofilaments; NT, neurotubules. Scale bar: 200 nm. **D** Transverse section of a population of large axons and surrounding extracellular space. MT, microtubules. Scale bar: 10 ${\upmu }$m. **E** Magnified structure of transverse extracellular space. VES, vesicles; N, glial nucleus. Scale bar: 0.5 ${\upmu }$m. **F** Longitudinal section of neck connective containing densely-packed microtubules and axons. Scale bar: 1 ${\upmu }$m. Abbreviations: A, axons; AM, actin microfilaments; D, desmosomes; G, ensheathing glia; M, mitochondria; MT, microtubules; N, glial nuclei; NAC, non-axonal cells; NF, neurofilaments; NL, neural lamella; NT, neurotubules; S, chromatin strands; T, tracheoles; V, vacuoles; VES, vesicles

Electron micrographs were obtained at the Emory University Robert P. Apkarian Integrated Electron Microscopy Core Facility. TEM images used in the primary mosaic of Fig. 1, higher magnification subpanels of Fig. 2, and the ensheathing glia survey of Fig. 4 were acquired using the JEOL JEM1400 TEM (JEOL, Japan). The SEM scan, shown in Fig. 1C, was acquired on a similarly sectioned and stained neck connective sample using a JEOL JSM-IT700HR SEM.

Two male *Manduca sexta* moths had their neck connectives sampled, epoxy embedded, and stained throughout this work. A male *Manduca* was used for the primary TEM mosaic of Fig. 1B. A different male was used for the SEM scan of Fig. 1C, the smaller TEM mosaic of Fig. 4, and all panels of Fig. 2. Some of these panel images involved additional sections, taken longitudinally down the length of the connective. Both TEM mosaics were acquired using semiautomated workflows with serial EM (Mastronarde 2005). The smaller initial mosaic was collected with 1996$\times$1992 pixel images at a resolution of 5.1 nm/pixel, while the larger complete mosaic was collected with 1980$\times$2024 images at the same 5.1 nm/pixel resolution.

**Fig. 3:**
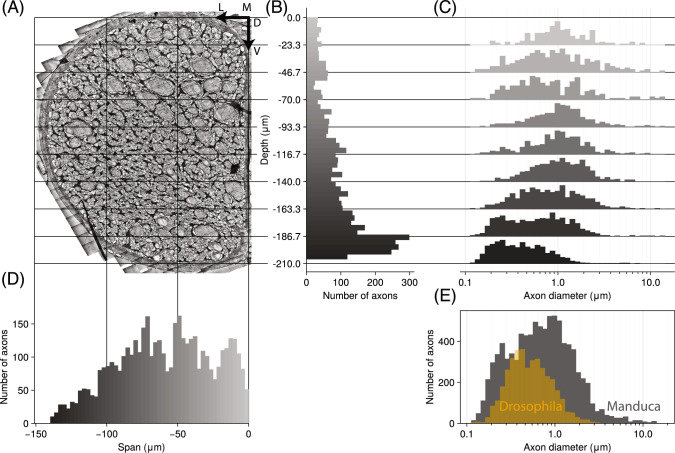
Distributions of axon positions and diameters in neck hemiconnective. **A** TEM mosaic, with gridlines corresponding to axes for plots in B, C, and D. Vertical axis is dorsal to ventral (D-V) depth, horizontal axis is medial to lateral (M-L) span. Note that vertical axis is shared by plots (**B**, **C**), and horizontal axis is shared with plot (**D**). **B** Distribution of axon centroids by depth, in ${\upmu }$m. Height of each bin corresponds to how many axons were located in that horizontal slice, shading indicates the distance from the origin of that bin. **C** Distribution of axon diameters for all axons contained within horizontal slices, with each slice defined by the region from the bottom of one histogram to the bottom of the histogram above it. Grey horizontal lines define these regions and carry across panels (**A**, **B**). Diameter distributions, on a log scale, are on the x axis. **D** Distribution of axon centroids by span, in ${\upmu }$m. Heights depict the number of axons in each vertical slice, shading indicates distance from origin of that bin. **E** Total distribution axon diameter across all axons, for *Manduca* (grey) and *Drosophila* (yellow). Axon diameter is on a log scale, and this axis is shared with (**C**)

The version of the main TEM mosaic shown in Figs. 1B and 3 was processed to improve large-scale visualization of the entire mosaic by evening contrast and brightness and reducing the visible effect of image stitching seams. Image processing was performed using Python 3.11.4 and Numpy (Harris et al. 2020). Images were first clipped so that the darkest 10% of pixels were brought up to a 10% brightness. This served to reduce the intensity of ultra-dark electron dense artifacts such as creases in the epoxy section. After clipping, flat-field correction was performed to remove any persistent brightness gradients or fixed-pattern noise by constructing a mean brightness map and removing that map from all images. If the mosaic is constructed from a set of *n* raw images defined as matrices $R_{i=1,\ldots ,n}$, flat field correction was performed by first obtaining an element-wise mean brightness value for each pixel

1 $$\begin{aligned} \bar{R}=\frac{1}{n}\sum ^n_{i=1}R_i. \end{aligned}$$

This mean brightness matrix $\bar{R}$ was normalized by its own mean $\mu _{\bar{R}}$, and then all images divided by the normalized mean brightness matrix to get corrected images

2 $$\begin{aligned} C_i = \frac{R_i}{\bar{R}/\mu _{\bar{R}}}. \end{aligned}$$

Each of these corrected images divided by the bright field were finally histogram equalized using contrast-limited adaptive histogram equalization from scikit-image’s exposure package 1.15.12 (Van der Walt et al. 2014) using a 1000 pixel kernel size. Final image export was performed using Pillow 11.0.0 (Clark 2015).

In addition to the TEM mosaics, a lower resolution but still complete SEM scan, shown in Fig. 1C, was acquired. This 21,360$\times$15,723 pixel image was acquired in single pass at a resolution of 12.28 nm/pixel. No additional processing was performed on this image after collection.

### Axon labeling and segmentation

To perform axon identification and segmentation, a U-Net model (Ronneberger et al. 2015) was trained. The training set for this model consisted of a manually segmented portion of the TEM image data (13 1996x1992 pixel tiles) where axons were identified by their characteristic high density of mitochondria and speckling pattern from microtubules, neurofilaments, and actin microfilaments. This training set was then enlarged with data augmentation consisting of horizontal and vertical shift and flips, shearing, and zooming. Image tiles were processed independently, to reduce the effects of brightness variation and other gradients present across the entire mosaic. Each tile within the mosaic was histogram equalized using ImageJ (Schneider et al. 2012), zero padded to $2048\times 0248$ and then split into $512\times 512$ patches for both training and prediction. For each patch, our U-Net would produce a probability that each pixel in the image is part of an axon. These probabilities were then thresholded at 0.5 to create a binary mask. Small objects within the mask with an area less than 400 square pixels (approximately 0.01 square microns) were then removed from the mask. The patches were then recombined and padding removed to create the automated prediction for each $1996\times 1992$ tile, and each of these images were then put together to get our prediction for the entire connective.

To perform proofreading on the automated segmentation, small chunks of the binary mask and corresponding TEM image were loaded into napari 0.5.3 (Chiu et al. 2022) and proofreading was performed on individual chunks before recombining them. Model accuracy varied greatly across different chunks, with some chunks being almost entirely correct and others requiring extensive proofreading work. After proofreading, any small segmentation artifacts were removed by once again removing objects with an area of less than 400 pixels.

The MorphoLibJ 1.6.4 (Legland et al. 2016) plugin for ImageJ was used to perform connected component labeling, getting a distinct label for each axon in our proofread segmentation. A Python script was used to extract information about the count, area, and centroids of each of the axons. As axons are not perfectly circular, diameters were calculated from axon area as the diameter of a circle with equivalent area, $d=2\sqrt{A/\pi }$, and are therefore lower bounds to the maximum edge-to-edge length.

A cross section of the neck connective of *Drosophila* was acquired from the MANC dataset (Takemura et al. 2023), accessed online through Neuroglancer. A z-level of 80,000 within the dataset was chosen for acquiring image data, and screenshots were taken at regular intervals to create a mosaic of the Drosophila neck connective. Images were sized such that each pixel within the image was 1.62 nanometers wide. Each image was later downscaled from $6831\times 3808$ to $1708\times 952$ through the GNU Image Manipulation Program (GIMP).

To segment the *Drosophila* neck connective, each individual tile was loaded into napari, and manual segmentation was done using Segment Anything for Microscopy 1.0.1 (Archit et al. 2025). Afterwards, tiles and their corresponding segmentations were put together and labels for axons split between different tiles merged to produce a segmentation of the entire mosaic.

In addition to the segmentation of whole hemiconnectives, a sample of 137 axons and their associated sheaths from the medial-dorsal portion of one hemiconnective were manually segmented in order to observe the scaling relationship between these structures using IMOD’s 3dmod program (Kremer et al. 1996). Although this sample is biased towards large axons given the location selected, this was our goal in order to receive sufficient data to compare the relative thicknesses of the sheaths surrounding small and large axons. In addition, the assignment of glial layers to their associated axons was determined by the following criteria:

- The ensheathing glia contacts the axolemma or glial membranes adjacent to the axolemma
- The ensheathing glia follows the general shape of the axon
- The ensheathing glia is not separated by any bands of dark, extracellular matter that consist of densely-packed microtubules

While this determination is difficult for small caliber axons, we attempt to normalize our findings by strictly adhering to the above criteria. Axon areas and the areas of axons plus their sheaths were calculated using trapezoidal integration from the set of (x, y) coordinates manually selected along the plasma membranes associated with that of the axon and the outermost layer of ensheathing glia. Diameters were calculated in the same way as described above for segmentation. Sheath thickness was determined by subtracting the fiber radius, meaning the radius of the axon and its surrounding sheath, and the axon radius.

### Conduction velocity measurements

To determine the maximum conduction velocities present in the *Manduca* neck connective, 4-shank, 4 electrodes per shank microelectrode arrays (Neuronexus A4x1-tet-3mm-150-121-A16) were used to take neck connective recordings at multiple simultaneous positions along the connective’s length. $N=6$ *Manduca sexta* moths were restrained in plastic tubing with their heads attached via fast-curing epoxy to a moveable platform. For each moth, the neck connective was exposed and held in place by a tungsten hook, and a microelectrode array inserted into the neck connective with all shanks penetrating the same side and approximate axial location on the hemiconnective. Recordings were acquired for 5–10 min before the probe was repositioned and a new set of axons recorded, with this process repeated 2–3 times per moth. All recordings were acquired with the Intan RHD2132 amplifier headstage using the Open-Ephys acquisition board and GUI (Siegle et al. 2017).

To identify putative neurons, or units, extracellular data was spike sorted using kilosort4 4.0.33 (Pachitariu et al. 2024). The unusual structure of many long axons oriented in parallel running across multiple shanks required some modifications on standard spike sorting parameters and preprocessing. High cross-channel correlations (with some spikes appearing on all 16 channels) mean that whitening or common average referencing can destroy some underlying spiking signals. For this reason, outside of standard high-pass filtering of all channels at 300 Hz using Spikeinterface 0.102.1 (Buccino et al. 2020), common average referencing was not performed, and the standard whitening in kilosort4 was modified. Instead of applying a 16$\times$16 whitening matrix *W* to the data, a whitening matrix weighted towards its diagonal (and thus only z-scoring channels) $W_{new}$ was used, defined as

3 $$\begin{aligned} W_{new} = \alpha W + (1-\alpha )W_{diag} \end{aligned}$$

where $W_{diag}$ is a form of the original whitening matrix where all non-diagonal entries are zero, and $\alpha$ is a weighting parameter which blends between a fully diagonal whitening matrix at $\alpha = 0$ (purely z-scoring) and normal whitening at $\alpha =1$. $\alpha$ was set to 0.1 for all recordings. Aside from preprocessing, parameters in kilosort were chosen to enable identification of units across multiple shanks. This includes providing the sorter a probe layout with all channels were on a single shank, with “shanks” placed 100 ${\upmu }$m closer to each other than reality, to enable template matching across multiple shanks. After spike sorting, all units were manually curated using Phy 2.6.0 (Rossant and Harris 2013; Rossant et al. 2025), so that only clearly defined units with no refractory period violations were included.

To measure conduction velocity, a cross-correlation method was employed similar to techniques used for sound source velocity and direction estimation from microphone arrays (Padois et al. 2017; Rhudy et al. 2009). The method simply involves measuring all time lags $\Delta t_{ij}$ and position offsets $\Delta x_{ij}$ between pairs of channels *i* and *j*. From these velocity is estimated as the mean of the resulting distribution of velocities $V=\mathbb {E}(\Delta x_{ij} / \Delta t_{ij})$. $\Delta x$ values come from the known distances between recording sites, and $\Delta t$ values are measured by finding the time lag between signals from recording sites at which the cross-correlation is maximized.

For each unit, 4 ms voltage waveforms were extracted from all channels centered on each occurrence of a spike, and the mean voltage waveform for each channel taken. Note these waveforms were extracted after bandpass filtering from 300 to 5000 Hz and z-scoring each channel, so all amplitudes are in units of standard deviations. If $x_i$ and $x_j$ are *n* samples long mean waveforms on the *i*th and *j*th channels, respectively, cross-correlation $c_{ij}$ across a range of time lags *l* were computed on all channel pairs as

4 $$\begin{aligned} c_{ij}[t] = \sum _{l=-n/2}^{n/2} x_i[t+l] \cdot x_j[t] \end{aligned}$$

where $x_j[t]$ denotes the value of mean waveform $x_j$ at sample *t*. This cross-correlation is computed across a range of time lags spanning the 4 ms spike length, for all pairs of channels (*i*, *j*) where both mean waveforms exceeded 1.5 standard deviations in amplitude and $\Delta x_{ij}$ was non-zero. To interpolate between samples and improve estimation of the time lag at which $c_{ij}$ is maximized, a quadratic is fit to the top 4 values of $c_{ij}$ and $\Delta t_{ij}$ is taken as the maximum of the quadratic. Note that sample times for each channel were shifted according to their actual acquisition time. While the recording was acquired at 30 kHz, the RHD 2132 actually operates 35 times faster, at 1.05 MHz, sampling each of its 32 channels (with 3 utility/pause samples) sequentially in a round-robin fashion. To compensate for this, each channel’s mean waveform was resampled according to its sampling order.

### Maximum likelihood estimation and statistics

To explore the relationship between distributions of axon diameters and conduction velocities, a Maximum Likelihood Estimation (MLE) technique was employed. For each value of *k* from a range of possible exponents linking axon velocity and diameter $v=d^k$, the likelihood of that *k* being explained by the measured data was calculated as follows: A theoretical set of diameters was calculated as $d_{est}=|v|^{1/k}$, and the probability density function (pdf) of $d_{est}$ determined via kernel density estimation. This pdf was used to evaluate the log of the probability of all experimentally measured values of *d*, and the sum of these taken to determine a log-likelihood value for that *k*. The *k* which maximized this log-likelihood was chosen as the most likely exponent given the data, and confidence intervals on this estimate were calculated using the bias-corrected and accelerated bootstrap interval. Bootstrapping was performed by repeating the main procedure 10,000 times, sampling velocities with replacement. All statistical tests performed here and throughout this work were performed in Julia 1.11.0.

## Results

### Anatomy of the neck connective

Since *Manduca* is bilaterally symmetric, we imaged only a single side of the two-sided neck connective. In *Manduca*, the two halves of the connective are separated and encased by connective tissue sheaths, called neural lamella. This is in contrast to dipterans (Coggshall et al. 1973), where the two halves are completely unified in the same lamella, and also differs from the fully separated left and right tracts in locusts (Burrows 1996; Williamson and Burns 1982). As is visible in Figs. 1B and 2A, the neural lamella is 4–5 ${\upmu }$m thick, several times thicker than what has been observed in flies (Coggshall et al. 1973), but on par with the $\sim$5 ${\upmu }$m lamellae in locusts (Rowell and Dorey 1967; Williamson and Burns 1982). The lamella in *Manduca* is interspersed with tracheoles (Fig. 1B) and is immediately followed by an internal layer of small ($\sim$1 ${\upmu }$m) non-axonal cells (Fig. 2A). Some tracheoles even make their way into the hemiconnectives, as observed in Fig. 2A.

Within the neck connective, all available space is primarily occupied by either axons or glia. An example larger axon of diameter 7 ${\upmu }$m is shown in Fig. 2B. As is common in electron microscopy of neural tissue, the axoplasm of neurons appears light grey at low magnification, but at very high magnification it is revealed that this is due to the staining of neurofilaments, actin microfilaments, and neurotubules (Fig. 2C), all of which are components of the neuron’s cytoskeleton (Bearer and Reese 1999; Hildebrand and Mohseni 2005). Axons also demonstrate an extremely high concentration of mitochondria, indicative of their high energy requirements. Despite the mosaic being a 70 nm ultrathin section, the density of mitochondria is high enough to catch typically dozens of mitochondria in any axon larger than 1 ${\upmu }$m. Some other standard cellular structures visible in axoplasm include vacuoles (appearing as light, unspeckled circular regions in Fig. 2A) and strands of chromatin (appearing as dark, string-like structures in Fig. 2B).

Using a convex hull of axon centroids to estimate total hemiconnective area, axons account for only 50.2% of the cross sectional area of the hemiconnective. The rest of this area is comprised primarily of glia, of which several qualitative types are apparent. One type ensheathes most axons larger than 1 ${\upmu }$m and is composed of many layers of plasma membrane. The other most prominent type of glia appears as heavily stained structures that fill in the spaces between axons (Fig. 2D). Upon close inspection, the dark medium of these interstitial glia are actually extremely close-packed microtubules with the occasional glial nucleus (Fig. 2E), identifiable via a characteristic cow-print pattern of euchromatin and chromatin differentially absorbing heavy metallic contrast (Cantera 1993; Cantera and Trujillo-Cenoz 1996). Longitudinal sections of the neck connective (Fig. 2F) confirm that these interstitial dense bundles of microtubules run along the length of the connective. While the function of these structures is not fully known, they have been observed in prior microscopy of the *Manduca* connectives (Pichon et al. 1972) and homologous structures are seen in other arthropods (Xu and Terakawa 1999). Such microtubule-rich processes are generally thought to serve a structural role. The connective and associated peripheral ganglia move a great deal during flapping, so it is reasonable to assume dense cables of microtubules could mechanically absorb forces, maintain structure, and provide support.

### Size, distribution, and overall number of axons

We identified and segmented a total of 4437 axons in one hemisection, indicating 8874 axons comprise the entire connective Fig. 3. For comparison, *Drosophila melanogaster* has 1328 descending and 2400 ascending neck connective neurons (Hsu and Bhandawat 2016; Namiki et al. 2018a; Stürner et al. 2025), and the desert locust *Schistocerca gregaria* has $\sim$ 6200 total axons (Rowell and Dorey 1967). Note the difference in body mass between these species: *Manduca* and *Schistocerca* typically weigh 1–3 g, but *Drosophila* orders of magnitude smaller at less than 1 mg.

The majority (by number) of axons in *Manduca*’s connective are located in the most ventral section (Fig. 3B). This is perhaps unsurprising given the clear spatial distribution of diameters present, with ventral axons on average much smaller (Fig. 3C) than medial or dorsal axons. For context, in flies what is known about spatial distribution of descending neurons indicates dorsal descending neurons are heavily associated with the wing neuropil, with ventral descending neurons associated with the leg neuropils (Namiki et al. 2018a). The overall distribution of axon diameters for *Manduca*, shown in Fig. 3E, is centered at a mean of 0.99 ${\upmu }$m and median of 0.69 ${\upmu }$m, and ranges from 0.1 to 15 ${\upmu }$m. While it may appear log-normal, the distribution of logged diameters $\ln (d)$ fails a Shapiro-Wilk normality test with $p < 0.001$. This is because the distribution is highly asymmetric, with a long tail of giant axons on the order of 10 ${\upmu }$m. The lack of normality may also be due to the clear spatial separation in axon diameters (Fig. 3C).

Alongside *Manduca*, the axon diameter distribution for *Drosophila*’s neck connective, gathered from the MANC dataset (Takemura et al. 2023), is provided in Fig. 3E. Compared to *Manduca*, *Drosophila*’s neck connective axons are on average smaller with a mean diameter of 0.61 ${\upmu }$m. Despite their more than order of magnitude contrast in body size, however, *Drosophila* does not have smaller axons than *Manduca*. Both species reach similar minimum axon diameters of 0.1 ${\upmu }$m, but *Drosophila* lacks a subpopulation of large axons outside of the 8 ${\upmu }$m giant fibers.

### Ensheathing glia

**Fig. 4:**
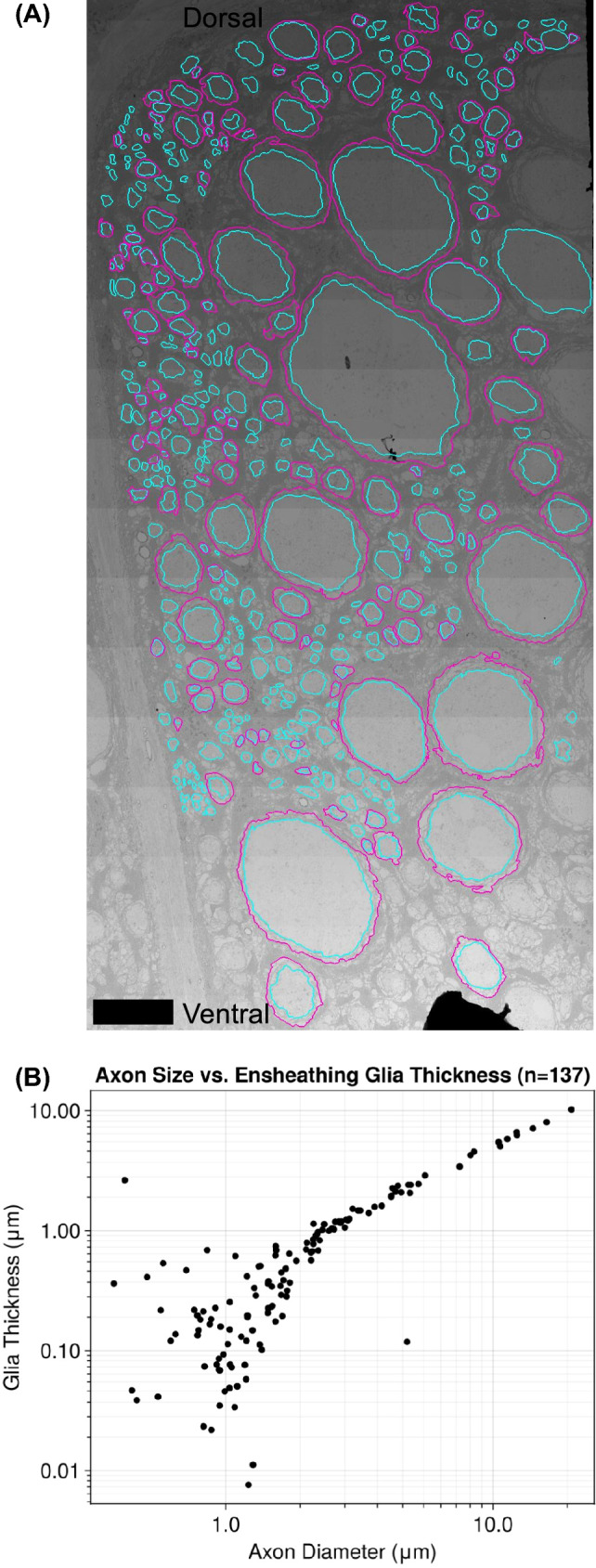
Ensheathing glia in the neck connective. **A** Illustration of the manually segmented sample of axons (n = 137), scale bar is 10 ${\upmu }$m. Teal, axons; magenta, glial sheaths. **B** Relationship between axon diameter and sheath thickness of the 137 axons and sheaths segmented

Axon sheaths are diverse structures, especially within the Arthropoda phylum (Bullock and Horridge 1965). This group of invertebrates go beyond the nonmyelinated and myelinated sheath categorization and have various distinct types, only one of which is truly considered myelin. On one side of the spectrum, axons can sport the expected nonmyelinated look, in which no glial cells or sheaths encompass the axon as is seen in the copepod *Eurytemora affinis* (Buskey et al. 2017) as well as some central nerves and most small peripheral nerves in Arthropods and other phyla (Bullock and Horridge 1965). On the other side, axons can be surrounded by thick amorphous coats alternating with layered glial processes, such as those observed in the wasp *Vespula carolina* leg (Edwards et al. 1958). Within this spectrum, there are various types of myelin-like glial sheaths that are characterized by less dense, more loosely wound layers of plasma membrane—like those wrapping *Drosophila* abdominal nerves (Kottmeier et al. 2020)—than true, compact myelin sheaths.

The glial sheath observed wrapping *Manduca* axons appears to lie within this range of myelin-like sheaths. Axons throughout the neck connective are surrounded by a sheath of loosely wound glial cells, which we refer to as ensheathing glia. These ensheathing glia have a lamellar structure, in which lipid bilayers are separated by the cytoplasm of these glial cells that appears white and amorphous. Adjacent layers of the plasma membranes are connected by desmosomes (Fig. 2B). Upon first glance, the ensheathing glia surrounding the axon seem to be arranged in a haphazard series of lamellae. However, after tracing the plasma membranes, it becomes apparent that the layers, of which we counted 5 on the axon pictured in Fig. 2B, are aligned concentrically around the axon. The amorphous nature of the ensheathing glia processes results in sections of the sheath being thicker than others despite an equal number of layers surrounding the axon at all points. As axon size increases for medium-large axons, however, the compaction of the sheath does as well, leading these axons to have sheaths resembling something more similar to densely wound myelin.

In order to explore the scaling relationship between axons and glial sheath thickness, 137 axons and their associated ensheathing glia were manually segmented while adhering to a set of criteria (Fig. 4A, B). Axons with diameters less than 2 ${\upmu }$m have no identifiable correlation between their size and the thickness of their corresponding sheaths because multiple small axons are frequently supported by single glial processes, leading to a large variation in sheath thickness for a given axon size. Conversely, medium to large axons greater than 2 ${\upmu }$m in diameter follow an isometric relationship with the thickness of their sheaths that can be described as $\log _{10}(t) = 1.12*\log _{10}(d)-0.45$. 95% confidence interval on the slope is $1.12 \pm 0.18$. No large axons are observed without dedicated ensheathing glia, and these glia evidently maintain nearly linear proportionality to the size of their axon.

### Conduction velocities

**Fig. 5:**
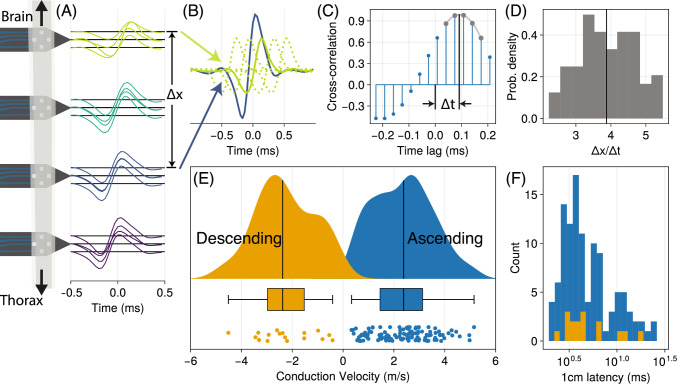
Conduction velocity measurement via multi-shank electrode array. **A** Scale diagram of the Neuronexus electrode array, with example mean voltage trace for each channel of a spike sorted unit. Shanks are to scale, with each shank 150 ${\upmu }$m apart. The mean voltage trace of an example unit for each channel is centered on a horizontal line placed at that channel’s position down the length of the connective. Mean voltage trace colors indicate shank. To calculate conduction velocity, a procedure diagrammed in panels (**B–D**) was followed. **B** Illustration of cross-correlation calculation. Between pairs of channels with detectable spikes, separated spatially by some distance $\Delta x$, one mean voltage trace (green) is time-lagged relative to another (blue), with correlation calculated at each time lag. Time lags are represented as dotted lines. **C** Cross correlation between mean voltage traces in (**B**) for range of time lags. Time delay $\Delta t$ for these two channels is determined as the lag of maximum correlation. To improve precision of this estimate, a quadratic is fit to the top 4 time lags, and the max of that quadratic used as $\Delta t$. **D** The procedure in (**B**, **C**) is repeated for all pairs of channels in (**A**) with detectable spikes, and the mean of all resulting $\Delta x / \Delta t$ values taken as the conduction velocity. **E** Conduction velocity for all units, across all $N=6$ moths, in m/s. Kernel density estimate, boxplot, and scatter of points are provided for descending (moving away from the brain) and ascending (moving toward the brain) units separately. **F** Conduction velocity data of (**E**) presented as milliseconds of latency for a 1 cm travel distance. Latency is on a log scale, colors are shared with panel (**E**)

Conduction velocities were measured using the multi-shank microelectrode array (Fig. 5A) and estimated using the procedure outlined in Fig. 5A–D. A total of 129 units across $N=6$ moths were observed (Fig. 5E), 112 of which were ascending (traveling towards the brain) and 17 of which were descending (traveling away from the brain). During manual curation, 24 of the ascending and 2 of the descending units were marked as potentially multi-unit activity; the rest were found to be of high certainty. Note that this is more ascending neurons than expected: While *Drosophila*’s neck connective has more ascending than descending neurons, the ratio is roughly $2\times$ as many ascending to descending (Stürner et al. 2025). It is important to emphasize that this preparation fully immobilizes the moth and does not involve targeted sensory stimuli. For these reasons, the activity recorded should be viewed as a biased sample, with little activity from neurons encoding motor activity or sensory information that was not present.

Both ascending and descending units had similar velocity distributions, with ascending units conducting at $2.42 \pm 1.2$ m/s and descending units at $-2.33 \pm 1.18$ m/s (mean ± 1 standard deviation). Ascending and descending velocity distributions are not significantly different in magnitude, failing to reject the null hypothesis in a non-parametric two-sample Kolmogorov-Smirnov test ($p=0.984$). The fastest and slowest units observed were both ascending, at 5.23 and 0.33 m/s, respectively. These values are within the observational bounds of this experiment of 13.5–0.23 m/s (set by sample rate, shank spacing, and spike time window), indicating that our measurements are not skewed or confined by experimental limitations. Conduction velocities are not normally distributed, with the distribution of all velocities (ascending and descending, |*V*|) rejecting a normal null hypothesis in a Shapiro-Wilk test ($p=0.02$). While not normal, the velocity distributions appear slightly bimodal, a property that might be a reflection of the distinct subpopulation of giant axons observed in the TEM mosaic (Fig. 3E).

Given the velocity distribution observed, and a rough estimate of *Manduca*’s neck connective as 1 cm in length, we provided the equivalent neck connective traversal latencies (Fig. 5F). Latency over a fixed length scales with $V^{-1}$ (note the log scale), so the resulting inverse distribution has heavier tails and greater spread. Mean latency is 6.35 ms across all units, with minimum and maximum 1 cm latencies of 1.94 and 30.11 ms, respectively. For context, in free-flight a single wingstroke from *Manduca* typically takes 40 ms (Gau et al. 2021), and the flight motor program of *Manduca* is temporally precise to the scale of 1 ms (Ortega et al. 2023). Some units are fast enough to cross from the brain to the thoracic ganglion in only $2\times$ the timescale of motor precision, while other units are slow enough to take almost a full wingstroke to traverse the neck connective. The velocities observed, then, are highly heterogeneous when considered as latencies, indicative of a nervous system where different types of information are given very different levels of priority. Given the bias of extracellular measurement towards the largest axons, our latency estimates of 2–30 ms should be considered a lower bound, with axons smaller than 1 ${\upmu }$m conducting much slower.

We have hypothesized that the temporal bottleneck of *Manduca*’s neck connective is particularly acute given the animal’s size, so adaptations to improve velocity and reduce latency are required. One mechanism for this is simply to develop larger axons (as seen in Fig. 3). But another way is to change axon physiology (such as increasing ion channel density) so that for the same diameter, a higher velocity is achieved (Castelfranco and Hartline 2015; Craner et al. 2004). The exponent *k* linking velocity and diameter, $v=d^k$, describes the degree to which these axons are conducting faster than a “typical” unmyelinated invertebrate axon, whose exponent should be $k=0.5$ (Hodgkin 1954; Rushton 1951). Ideally, this scaling exponent would be directly estimated via regression, but our conduction velocities and diameters were measured independently of each other. Each velocity measurement does not come with a known diameter, and vice versa. Instead, we estimated the exponent *most likely* to link the measured velocity and diameter distributions by calculating the log-likelihood that a given value of *k* explains the data observed, across a range of $k\in [0.4,1.5]$. Only diameters greater than 3 ${\upmu }$m are used, based on the *a priori* assumption that velocity measurements were limited to axons of that size. This analysis determined the most likely exponent given the data observed is $k=0.64$ (95% confidence interval: [0.614, 0.652]), a value significantly higher than the standard invertebrate scaling.

## Discussion

### *Manduca* has an abundance of giant axons, separated into clear spatial subpopulations

Alongside *Manduca*, the axon diameter distribution for *Drosophila*’s neck connective, gathered from the MANC dataset (Takemura et al. 2023), is provided in Fig. 3E. Compared to *Manduca*, *Drosophila*’s neck connective axons are on average smaller with a mean diameter of 0.61 ${\upmu }$m. Despite their more than order of magnitude contrast in body size, however, *Drosophila* does not have smaller axons than *Manduca*. Both species reach similar minimum axon diameters of 0.1 ${\upmu }$m, but *Drosophila* lacks a subpopulation of large axons outside of the two 8 ${\upmu }$m giant fibers. For small axons, both species are bounded by the established lower limit of axon diameter of 0.1 ${\upmu }$m, below which ion channel thermal noise causes spontaneous firing (Faisal et al. 2005). But on the higher end, demands on *Manduca* for faster conduction have clearly pushed some neck connective neurons towards gigantism, with 22 axons per hemiconnective greater in diameter than *Drosophila*’s giant fiber and 68 axons greater than 5 ${\upmu }$m in diameter. While there is a lower limit on axon size, there is no physical upper constraining limit other than the metabolic costs for supporting giant axons and packing limits within the cross-sectional area of the connective. Given *Manduca*’s neck connective is quite small in relative to the rest of its neck, metabolic costs are likely the only main factor constraining axon gigantism.

The giant axons in the *Manduca* neck connective separate into some clear populations based on their spatial distribution. From Fig. 3, as well as cursory observation of Fig. 1B, C, it is clear that the largest axons are distributed either in a large dorsal population or in a smaller ventral population, with a few scattered around the midline. This matches previous coarser imaging of the neck connective of *Manduca* (Gray et al. 2002; Kanzaki et al. 1991). Such patterning may be indicative of a high importance for low latency placed on flight circuits in particular. While less is known about spatial distributions of ascending neurons, in *Drosophila* dorsal descending neurons are heavily associated with the wing neuropil, with ventral descending neurons associated with the leg neuropils (Namiki et al. 2018a). There is evidence that descending neurons in lepidopterans and dipterans are reasonably homologous (Namiki et al. 2018b; Hsu and Bhandawat 2016), indicating the dorsal and ventral populations of large neck connective neurons present here are associated with flight and walking, respectively. It is reasonable to speculate that the abundance of the smallest neurons in the neck connective in the ventral portion, and the shift towards much larger neurons in the dorsal region (see Fig. 3), may simply be consequences of the life history of adult *Manduca* involving more flight than walking.

### *Manduca*’s neck connective is similar in number to insects of very different brain size

We identified 4437 axons in a single side of *Manduca*’s neck connective, indicating $\sim$ 8800 total neurons in the neck connective, roughly twice the $\sim$3700 neck connective neurons of *Drosophila*. While the number of neurons in *Manduca*’s brain has not been estimated before, *Hyles lineata*, a closely related hawkmoth with similar life history and slightly smaller in body mass than *Manduca*, has been measured to have an adult brain of $\sim$ 1 million neurons (Aksamit et al. 2024). Assuming *Manduca* has a similar brain to *Hyles*, this indicates an order of magnitude more neurons in the brain than *Drosophila*, yet only twice as many neurons in the neck connective. The desert locust *Schistocerca gregaria*, one of the only other insects to have a neck connective fully surveyed, had $\sim$ 6200 total axons (Rowell and Dorey 1967). *Schistocerca*’s adult body mass is slightly larger than *Manduca*, 2–3 g (Fischer and Kutsch 2000), so it may be unsurprising that it has a similarly sized neck connective. Hsu et al. have suggested as much before when comparing descending neurons from backfill studies, noting that most arthropods studied have highly homologous descending neurons in organization and count, despite hundreds of millions of years of evolutionary separation (Hsu and Bhandawat 2016).

This apparent parsimony in neck connective size in insects may have a few possible explanations. For one, much of the scaling of nervous systems is tied to body mass via the size of sensory and motor structures (Chittka and Niven 2009). For instance, while *Hyles lineata* has a brain of $10^6$ neurons while *Drosophila* only $10^5$, 60% of those neurons are simply in the optic lobe, handling the processing of larger eyes with more facets. By virtue of being several layers of computation past primary sensory inputs, neck connective neurons may simply be carrying higher-level features of vision, locomotion, and other information that are relatively invariant to the size of the animal. A motor-oriented version of this hypothesis has been suggested before, that the highly reduced number of descending neurons in invertebrates compared to vertebrates may be due to the extremely high number of motor neurons used by vertebrates (Hsu and Bhandawat 2016). Certainly, known insect descending neurons tend to represent larger features. Target-selective descending neurons integrate both small- and wide-field visual features (Nicholas et al. 2018), and specific descending neurons are known that tune large-scale features of locomotion in walking (Yang et al. 2024) and flying (Ros et al. 2024). This kind of feature extraction inherent in so many aspects of neural computation is compressive, and this compression may be enough that comparatively little more information is actually needed to control a bigger animal than a smaller one.

Another possible explanation may be in the potential energetic cost of neck connective neurons. The cost just to maintain a polarized membrane is in some cases 13% of the energetic cost of a neuron’s activity (Attwell and Laughlin 2001), and this cost scales linearly with axon diameter and axon length. It may simply be energetically advantageous to reduce both the size and number of connective tract axons. Note this is not mutually exclusive with the previous theory of the neck connective being smaller due to feature extraction, and may even be complementary.

### Ascending and descending units are similarly fast

It is easy to assume a strong hierarchy in the nervous system where the brain is heavily feedforward and prescriptive of an animal’s actions, and thus descending signals are especially high-priority. This is partially why descending neurons have been the object of so much more study than ascending neurons in insects (Hsu and Bhandawat 2016; Liu et al. 2023; Namiki et al. 2018b; Okada et al. 2003; Severina et al. 2016; Staudacher 1998). But our results indicate that, of the large axons detected via extracellular probe, there is no distinction in the velocity (and therefore latency) of ascending or descending axons (Fig. 5E, F). Certainly by axon number alone, the neck connective of *Drosophila* is actually far more dominated by ascending neurons, with $2\times$ as many ascending as descending neurons (Stürner et al. 2025). If the assignment of energetically costly large, fast axons is indicative of relative priority, the ascending signals in *Manduca*—likely composed of sensory information, motor efference copies, and other indicators of body state—are apparently treated with similar priority to descending commands.

However, the extracellular sampling of neurons as we have done here is a biased sampling, so interpretation of the relative number of ascending vs. descending units is difficult. Extracellular electrodes preferentially observe the largest (and therefore fastest and electrically “loudest”) axons, but are not a uniform sampling of all available axons. We are also limited to only observing axons that fire enough to be identifiable. In a tethered preparation, with little variation in visual stimuli as we have performed here, many descending units conveying visual motion, flight motor commands, or other signals may simply not be sampled, or too sparse to have been identified by our spike sorting.

### Ensheathing glia may lead to faster, but not saltatory, conduction

The many-layered glia ensheathing *Manduca*’s largest axons (Figs. 2, 4) are of particular note in part due to their resemblance to myelin. Myelin, glia which encircle axons with many layers of membrane, is ubiquitous in vertebrates, has independently evolved at least 3 times (Castelfranco and Hartline 2015; Hartline 2008) and is observed in arthropods, annelids, and even the leg nerves (but not the neck connective) of *Drosophila* (Kottmeier et al. 2020; Rey et al. 2023). The fact that the ensheathing glia seen in this study preferentially surround all large axons (Fig. 4) suggests convergent function. Moreover, in *Manduca* such multi-layered sheaths have not been observed in the brain or peripheral ganglia (Cantera 1993). In other insects, these ensheathing glia seem to be present only in very long tracts, such as the leg nerves of *Drosophila* (Kottmeier et al. 2020; Rey et al. 2023), large neck axons of locusts (Rowell and Dorey 1967), or leg motor neurons of cockroaches (Pearson et al. 1970), and absent in shorter tracts such as the neck connectives of smaller dipterans (Coggshall et al. 1973). These patterns may simply be borne out of ensheathing glia serving functions preferentially needed by large, fast axons such as energy support or reduction of ephatic coupling. But given our hypothesis that *Manduca* faces high pressures on neck connective latency, it is worth exploring whether these glia serve to improve conduction velocity.

To increase conduction velocity and functionally serve as myelin, any ensheathing glia must meet two criteria: (1) Greatly decrease effective membrane capacitance of the axon-glia fiber and increase fiber resistance, and (2) Have periodic breaks exposing the axon’s membrane to extracellular space called “nodes”, e.g. nodes of Ranvier (Hartline 2008; Xu and Terakawa 2013). The first criterion increases the velocity by changing the space and time constants of action potential propagation. The second criterion, of exposed nodes, forces that propagation to be *saltatory*, so current from one depolarizing region must jump to adjacent nodes rather than exhaust itself locally. Much of the conduction velocity increase from myelin is due to this second criterion, but resistance and capacitance changes resulting from the presence of myelin sheaths alone still greatly affect velocity. Interestingly, most of these effects are due to myelin decreasing effective membrane capacitance: Halving the capacitance of an unmyelinated axon increases velocity by 50%, whereas halving the membrane resistance only produces a 2% gain (Hartline 2008). The increase in effective membrane resistance from myelin actually slightly decreases conduction velocity, but this is far outweighed by both capacitance changes and the fact that increased insulation forces saltatory conduction. All of these properties require myelin to tightly encircle axons, leaving no path for ions to simply bypass the myelin and remove any benefits conveyed. The anatomical form by which this is accomplished varies from tight spirally-wrapped membranes in vertebrates and annelids (Hartline and Colman 2007) to loose concentric layers in shrimp (Xu and Terakawa 2013), but all forms of myelin meet the criteria listed.

By these criteria, *Manduca*’s ensheathing glia are not myelin because no nodes, or sections of exposed axon membrane, were observed in any EM images. This includes longitudinal sections taken explicitly to search for nodes (Fig. 2F). It is possible that our sections may have simply failed to intersect with any nodes. Typically even non-myelin ensheathing glia do not extend the full length of the axons they ensheath, inherently leaving some exposed regions of membrane (Castelfranco and Hartline 2015; Fernandes et al. 2024); that those natural ends were not observed indicates our longitudinal sections were limited. Regardless, in this study of *Manduca*, no exposed regions of any kind were observed, so saltatory conduction is most likely not present.

However, while we did not observe regular nodes, ensheathing glia form a spectrum of myelin-like properties, of which true myelin is simply one extreme (Bullock and Horridge 1965). An evolutionary intermediate to myelin, where ensheathing glia impede the flow of current from an axon, still increases velocity by decreasing the capacitance of the axonal membrane (Castelfranco and Hartline 2015; Hartline and Colman 2007). Typical wrapping glia that surround axons are thought to impede the flow of current very little, as gaps of 20 nm or less are sufficient for current to escape, even when axons are surrounded by several layers of glia (Binstock et al. 1975; Castelfranco and Hartline 2015). As such, for any ensheathing glia to impact conduction, it must actually alter the flow of ions. Most myelin accomplishes this via tight spiral wrapping so that the path to extracellular space is both long and extremely narrow. But an alternative method of myelination, seen in *Penaeus* shrimp, is to *concentrically* surround axons, forcing the path to ground along the much farther length of the neck connective from the brain to the peripheral ganglia, rather than the radius of the axon-glia fiber. This enables fewer, looser layers of membrane to provide the same decreased capacitance and increased resistance (Xu and Terakawa 2013). Ensheathing glia in *Manduca* have this concentric property (Fig. 2B); while no axon is surrounded by more than 10–15 layers of membrane, all of these layers surround the axon concentrically as in *Penaeus* shrimp, so that gaps to extracellular space must happen only at the ends of the ensheathing glia. This anatomy suggests that *Manduca*’s ensheathing glia may be impacting conduction.

As our maximum likelihood estimation showed, the most likely scaling between axon velocity and diameter in *Manduca*’s neck connective is $k=0.64$ (95% confidence interval: [0.614, 0.652]). This scaling is in between the linear $k=1$ scaling of myelin and the $k=0.5$ scaling of typical invertebrate unmyelined axons. Scaling exponents of 0.7$-$0.8 have been observed before in insects, notably in cockroach leg motor neurons and the locust tergal nerve (Pearson et al. 1970). Pearson et al. in particular were unable to resolve why their measured scaling was faster than expected, but it is noteworthy that images included in their paper show concentric, many-layered ensheathing glia around the cockroach motor neurons recorded. While faster scaling can be explained by large increases in ion channel density (Castelfranco and Hartline 2015; Craner et al. 2004), that approach incurs high energetic costs across large, long axons (Attwell and Laughlin 2001). Instead, we propose that the ensheathing glia observed here may be sufficient to provide the speedup. While the ensheathing glia in *Manduca* are likely not providing saltatory conduction, they may enhance conduction velocity through the mechanisms described, accounting for the intermediate scaling values observed in this paper and other large insects.

There is no doubt that the ensheathing glia observed here serve many likely roles, including preventing large axons from ephatically coupling and potentially blending their signals (Kottmeier et al. 2020; Nave and Werner 2021) or separating axons and neural tissue from *Manduca*’s high-potassium hemolymph (Pichon et al. 1972). Myelin’s role in reducing the energetic cost of axons is extremely noteworthy (Perge et al. 2009), and myelin is suggested to have originally evolved for conserving energy first, with saltatory conduction only developed later (Stiefel et al. 2013). The ensheathing glia present in *Manduca* may be on this evolutionary trajectory, increasing conduction with less energetic cost than other options. Further work, however, is necessary; while the evidence presented here is sufficient for us to propose a role for ensheathing glia to improve conduction in *Manduca*, actual manipulations and measurements of the electrical and energetic properties of these glia are required to confirm this prediction.

### Is the neck connective a bottleneck in *Manduca*?

We originally hypothesized that large flying insects like *Manduca* have a large spatial bottleneck, opting for far fewer connective neurons than their brain size would suggest. This was confirmed: *Manduca* has at least $100\times$ fewer neurons in the neck connective than the brain, and the absolute number of neurons in its neck connective is not, in absolute terms, very different from what is observed in both larger and smaller insects.

While the neck connective is certainly a spatial bottleneck in terms of the number of neurons, it is more difficult to gauge whether this really is a bottleneck of information capacity. The information rate of neurons varies greatly based on their firing rate, temporal precision, and the signals they encode, but generally varies from 10 to 200 bits/s (Borst and Theunissen 1999). Assuming even a very conservative estimate of average information rate at $\sim$5 bits/s, across 8800 neurons that still produces a neck connective carrying $\sim$40–50,000 bits/s of information. While this seems a paltry figure in comparison to the 1.5 Mbits/s of even a 1996 USB 1.0 cable, how much information does an insect brain need to understand the sensory state of its body and effectively control itself? 40–50,000 bits, every second, is an immensity of possible states that can be represented, and very well may be plenty. Thus, while the *Manduca* neck connective is *numerically* a bottleneck, and certainly requires the brain to compress some sensorimotor information into smaller forms, likely with significant information loss, it may not be a substantial informational bottleneck.

We also hypothesized that *Manduca*’s size makes the temporal bottleneck, in terms of signal latency, quite acute, and that these insects would reduce this limitation through enhanced conduction velocities via giant axons or other adaptations. Our observations matched this hypothesis: We observed multiple spatially separate populations of giant axons, and 30+ axons per hemiconnective larger than *Drosophila*’s giant fiber (Fig. 3). These large axons are both ascending and descending, conducting at velocities on the order of 2–5 m/s (Fig. 5). The presence of so many large, energetically costly axons is certainly indicative of pressures on *Manduca* to minimize neck connective latency. As discussed, the conduction velocity of these axons is also indicative of potential adaptations for increased velocity, including ensheathing glia.

We observed velocities with equivalent latencies of 2 to 30 ms (Fig. 5). 96% of *Manduca*’s connective axons, however, are smaller than 3 ${\upmu }$m (Fig. 3E) and likely not observed extracellularly. This suggests the vast majority of information would take more than 30 ms to traverse the neck connective. A single wingstroke takes 40 ms, so much of the information traveling between the brain and the body is likely a full wingstroke behind. Evidently *Manduca* has far more heterogeneity in signal propagation time than animals like *Drosophila* (Fig. 3). Some ascending and descending signals with high priority on latency can cross the neck connective in single milliseconds, but most other information takes far longer. As with the spatial bottleneck, whether this constitutes a temporal bottleneck needs context and consideration of what latency would be limiting for an animal to function. In mammals, conduction delay is up to 40% of total sensorimotor delay (More and Donelan 2018), though while this is most acute in larger mammals, these increasing conduction delays are generally offset by longer movement durations (More et al. 2010). There is evidence that *Manduca* is capable of responding to perturbations on the timescale of 2–4 wingstrokes (Gau et al. 2021), and its flight motor output is temporally precise to a sub-millisecond scale (Ortega et al. 2023). Clearly large hawkmoths have measurable temporal limitations. Further work, however, is needed to determine if these and other temporal limits in motor output generation, perturbation responses, and other behaviors are limited by conduction delays, or by other factors like mechanics inherent to the flight motor system, or upstream and downstream circuits on either side of the neck connective.

## Data Availability

Electron microscopy images and their associated data, as well as conduction velocity data, are available on GT SMART repository. Code for all data analyses and figure generation can be found at https://github.com/LeoJW/JCPA2025.
